# Effects of PPARγ in dendritic cells during severe sepsis and sepsis-induced immunosuppression

**DOI:** 10.1186/cc13000

**Published:** 2013-11-05

**Authors:** Raphael Molinaro, Roberta Navarro-Xavier, Papp Attila, Adriana Vieira-de-Abreu, Adriana Ribeiro Silva, Hugo Caire Castro-Faria-Neto, Claudia Farias Benjamim, Laszlo Nagy, Patrícia Torres Bozza

**Affiliations:** 1 Immunopharmacology Laboratory, IOC/FIOCRUZ, RJ, Brazil; 2 Nuclear Hormone Receptors Laboratory, Debrecen, Hungary; 3 Inflammation Laboratory, UFRJ, Brazil

## Background

Sepsis is a systemic inflammatory response syndrome against infection, which can develop in sepsis-associated immunosuppression. Actually, several inflammatory dysfunctions have been described in dendritic cells (DCs) that could be responsible for impairing the immune response towards the secondary infection. PPARγ is a lipid-activated nuclear receptor, which participates in inflammation, lipid metabolism and cellular differentiation. Previous studies have shown the role of PPARγ in acute sepsis besides its effects in sepsis-induced immunosuppression still being unclear. Our aims were to evaluate the phenotypic changes in DCs in lungs from post-septic mice and to assess the effects of PPARγ on DC functions.

## Materials and methods

Mice were subjected to cecum ligation and puncture (CLP) or Sham and, 6 hours after, all groups were treated with antibiotics. Fourteen post-septic and Sham mice were infected with BCG and 24 hours after challenge the lungs were collected, minced and digested to investigate the cytokine production, gene expression and phenotype analysis. To evaluate the effects of PPARγ, post-septic derived BMDC were pretreated with PPARγ agonist (rosiglitazone) before BCG infection. After 24 hours, lipid droplet formation, phagocytosis, cytokines and oxide nitric production were analyzed.

## Results

Post-septic mice were susceptible against *Mycobacterium bovis*, BCG and exhibited higher cellular infiltration. Lungs from post-septic mice showed increased IL-10 level and COX2, CCR2 and IL-1β expression. When post-septic and Sham mice were infected with BCG, we observed higher increased COX2, CCR2 and IL-1β expression in lungs from post-septic mice as compared with lungs from Sham mice but the IL-10 level was reduced. In addition, lungs from post-septic mice showed higher Ly6G cells compared with lungs from Sham mice. Infected BMDC exhibited an immature profile (lower expression of CD80 and CD40) and a positive shift to anti-inflammatory cytokine production (increased IL-10 and reduced TNFα, CCL2 and IL-1β levels). PPARγ flanked mice in CD11c cells were more susceptible to severe sepsis. Activation of PPARγ in infected BMDC from post-septic mice reduced lipid droplet formation, phagocytosis and oxide nitric production but not cytokine production when compared with infected BMDC from Sham mice.

## Conclusions

After severe sepsis, phenotypic changes modulate DC functions and may contribute to sepsis-induced immunosuppression. The understanding of PPARγ could be important for development of new therapy in sepsis-associated immunosuppression and long-term inflammatory diseases.

